# SARS-CoV-2: From Structure to Pathology, Host Immune Response and Therapeutic Management

**DOI:** 10.3390/microorganisms8101468

**Published:** 2020-09-24

**Authors:** Grigore Mihaescu, Mariana Carmen Chifiriuc, Ciprian Iliescu, Corneliu Ovidiu Vrancianu, Lia-Mara Ditu, Luminita Gabriela Marutescu, Raluca Grigore, Șerban Berteșteanu, Marian Constantin, Gratiela Gradisteanu Pircalabioru

**Affiliations:** 1 Microbiology Immunology Department, Faculty of Biology, University of Bucharest, 050095 Bucharest, Romania; mihaescuapelevii@gmail.com (G.M.); ovidiu.vrancianu@yahoo.com (C.O.V.); lia_mara_d@yahoo.com (L.-M.D.); lumi.marutescu@gmail.com (L.G.M.); gratiela87@gmail.com (G.G.P.); 2 Research Institute of the University of Bucharest, 050095 Bucharest, Romania; 3 National Institute for Research and Development in Microtechnologies—IMT, 077190 Bucharest, Romania; cipi_sil@yahoo.com; 4 Academy of Romanian Scientists, 010071 Bucharest, Romania; 5 ENT Department, University of Medicine and Pharmacy Carol Davila and Coltea Clinical Hospital, 020022 Bucharest, Romania; raluca.grigore@3f.ro (R.G.); berstein@dr.com (Ș.B.); 6 Institute of Biology, 060031 Bucharest, Romania; cvgmarian@gmail.com

**Keywords:** SARS-CoV-2, COVID-19, immune response, inflammation

## Abstract

Coronaviruses are large, enveloped viruses with a single-stranded RNA genome, infecting both humans and a wide range of wild and domestic animals. SARS-CoV-2, the agent of the COVID-19 pandemic, has 80% sequence homology with SARS-CoV-1 and 96–98% homology with coronaviruses isolated from bats. The spread of infection is favored by prolonged exposure to high densities of aerosols indoors. Current studies have shown that SARS-CoV-2 is much more stable than other coronaviruses and viral respiratory pathogens. The severe forms of infection are associated with several risk factors, including advanced age, metabolic syndrome, diabetes, obesity, chronic inflammatory or autoimmune disease, and other preexisting infectious diseases, all having in common the pre-existence of a pro-inflammatory condition. Consequently, it is essential to understand the relationship between the inflammatory process and the specific immune response in SARS-CoV-2 infection. In this review, we present a general characterization of the SARS-CoV-2 virus (origin, sensitivity to chemical and physical factors, multiplication cycle, genetic variability), the molecular mechanisms of COVID-19 pathology, the host immune response and discuss how the inflammatory conditions associated with different diseases could increase the risk of COVID-19. Last, but not least, we briefly review the SARS-CoV-2 diagnostics, pharmacology, and future approaches toward vaccine development.

## 1. Introduction

Coronaviruses are large, enveloped viruses, having a spherical morphology, although they can often be pleomorphic, with a single-stranded RNA (ssRNA) genome of positive polarity. Coronaviruses have prominent glycoprotein spikes and infect both humans and a wide range of animal species. The first coronaviruses (E229 and OC43) were described by Hamre et al. (1966) in patients with upper respiratory tract infections. Notably, healthy carriers of coronaviruses represent approximately 2% of the human population [1]. Coronaviridae family is classified into two subfamilies: *Letovirinae* and *Orthocoronavirinae*. The subfamily *Orthocoronavirinae* includes *α-*corona-, *β-*corona-, *γ-*corona-, and *δ*-coronaviruses [2]. Of the four genera, the first two infect only mammals (palm owl, monkeys, cat, ferret, hamster, raccoon dog, and especially bats). The last two primarily infect birds but have also been isolated from pigs.

Seven types of coronaviruses infect humans: four of them (E229 and OC43, HKU1 and NL63) are agents of seasonal infections (shCoV), which became globally endemic causing 2–18% of the human common colds during the winter months, but also gastrointestinal disorders and bronchiolitis, even pneumonia, in immunodefficient patients [3]. They confer cross immunization in children up to four years of age. The last three coronaviruses infectious for human belong to the order *Nidovirales* and are SARS (severe acute respiratory syndrome) agents, causing infections with a severe clinical picture and high mortality; these three coronaviruses are SARS-CoV (possible intermediary host, *Civetta civettictis*), MERS (Middle East respiratory syndrome, originating in Saudi Arabia, possible intermediary host—camel), and SARS-CoV-2—possible intermediary host—*Manis javanica* or pangolin [4]. OC43, HKU1 and all the three novel SARS coronaviruses belong to β-coronaviruses [5].

The extensive animal reservoirs, high mutation rate, and genetic recombination are predisposing features for *Coronaviruses* to easy jump to different hosts. All three coronaviruses causing SARS in humans share at least one cellular receptor, such as the angiotensin-converting enzyme or angiotensin-converting enzyme II (ACE2) which interacts with the S (spike) glycoprotein inserted in the viral peplos. The same ACE2 receptor mediates both SARS-CoV and SARS-CoV-2 interaction with the human and animal reservoirs cells expressing this receptor. MERS-CoV binds to sensitive cell membranes, primarily by dipeptidyl-peptidase 4 (DPP4), and secondarily by ACE2 [6]. Another unifying factor facilitating the interspecific spread is the tissue protease - furin, which cleaves the S glycoprotein, mediating the fusion of the viral spike with the ACE2 receptor, followed by the passage of the viral nucleocapsid into the host cell cytoplasm) [7].

In this review, we will first present a brief description of the SARS-CoV2 virus, discuss the host immune response in COVID-19, the mechanisms explaining the increased risk of different populations, and the current approaches in diagnosis, treatment and prophylaxis by vaccination.

## 2. The Molecular Structure and Origin of SARS-CoV-2

**Figure 1:**
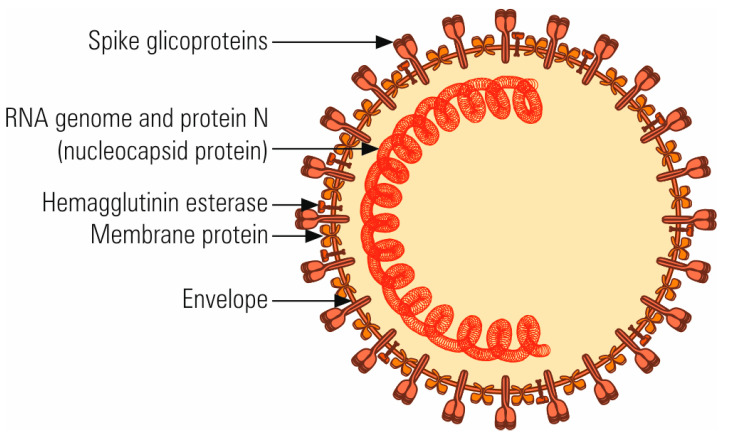
Schematic representation of virion molecular structure. The S spikes are most prominent on the virion surface and confer the specific appearance of corona. The transmembrane M protein is highly hydrophobic, with three domains in the peplos. The E glycoprotein is a minor constituent of peplos. Some coronaviruses have an additional protein, HE (haemagglutinin-esterase).

The size of SARS-CoV-2 ranges between 60–140 nm due to its pleomorphism. The virion is enveloped by peplos derived from the endoplasmic reticulum or Golgi cisternae membranes. The peplos is crossed by prominent S spikes (Figure 1) and by other glycoproteins (E, M). The peplos covers the virion central body, represented by the nucleocapsid which contains the genome and the phosphorylated nucleoprotein (N). The genome size ranges between 26–32 kb.

The SARS-CoV-2 genome sequence shares 80% homology with the SARS-CoV virus and 96–98% with the virus isolated from bats [8]. SARS-CoV infects bats, and *Civetta civettictis* acts as the intermediate host, from which it passed to humans [7]. Although the World Health Organization (WHO) states that the transition of SARS-CoV-2 from an animal host to humans remains uncertain, a hypothesis has been formulated. Due to the high homology with a coronavirus isolated from bats and, to a lesser extent, with a virus isolated from pangolins, it is thought that the pangolin could be the intermediate host mediating the transmission of SARS-CoV2 from bat to man. The receptor-binding domain (RBD) of the spike S ligand contains the same six amino acid residues in SARS-CoV-2 as in the virus isolated from pangolin. However, at genomic level, the virus isolated from pangolins is not perfectly similar to SARS-CoV-2, lacking a polybasic site sensitive to the action of furin [9]. 

## 3. Sensitivity to Physical and Chemical Agents

Both SARS-CoV-1and SARS-CoV-2 viruses are stable and infectious in aerosols for several hours, and on surfaces, up to several days, depending on the density of the contaminant inoculum. Interhuman transmission is mainly achieved through infected respiratory secretions, eliminated by coughing or sneezing in the form of aerosols. The spread of infection is favored by prolonged exposure to high densities of aerosols indoors. Direct contact with an infected hand or a contaminated surface, followed by reflex movements to touch the oral cavity, nasal cavity, or ocular conjunctiva, are also possible routes of transmission. Nevertheless, despite consistent evidence as to SARS-CoV-2 contamination of surfaces and its survival on certain surfaces, there are no specific reports which have directly demonstrated fomite transmission [10]. Fecal-oral transmission is possible, but fecal isolation of SARS-CoV-2 has been rarely reported [11]. It has been observed that SARS-CoV-2 can preserve its ability to infect for approximately two weeks under temperature and humidity conditions found in an air-conditioned environment. SARS-CoV-2 remains infectious for at least five days at a temperature of 22–25 °C and a humidity of 40–50%. The loss of infectiousness occurs at temperatures above 38 °C and humidity above 95% [12,13,14]. These data demonstrate that although direct droplet transmission is the main route of contamination, contaminated surfaces could also be a possible route of viral transmission. Furthermore, it has been observed that exposure to UV radiation and temperatures above 56 °C causes the inactivation of the virus [15]. Coronaviruses are also sensitive to lipid solvents (ether, 75% alcohol), chlorine, peroxyacetic acid, and chloroform disinfectants, but not to chlorhexidine [16]. Currently, studies have shown that SARS-CoV-2 is considerably stable when compared with other types of coronaviruses, as well as other viral respiratory pathogens. These findings necessitate the implementation of increased safety measures, given that SARS-CoV-2 could present alternative routes of transmission, in addition to the main route through droplets.

## 4. Viral Infection Cycle

### 4.1. Virus-Cell Interaction

The apical pole of epithelial cells is lined with protective glycocalyx layer (400–500 nm), in which hydrolytic enzymes are found. To bind to membrane receptors, viral spikes with mucopolysaccharidase activity hydrolyze the mucus barrier. 

The tropism of several viruses for specific cells or tissues, and implicitly the pathological manifestations, are determined by the specificity of the interaction between the viral ligand and cellular membrane receptor. 

The common cold coronaviruses, but also influenza virus, interact with the sialic acid or other carbohydrates via the carbohydrate component of the viral S glycoproteins. In order to infect a cell, a critical density of membrane receptors to interact with a sufficient number of ligands on the surface of the virion is required. The S glycoprotein of SARS-CoV-2 is a homotrimer with 71% amino acid sequence homology to SARS-CoV and 97% to S protein of RaTG13 virus isolated from bat. These facts explain the differences in the affinity of the two SARS viruses for the cellular receptors and in the efficiency of viral infections propagation.

The main cellular receptor for SARS-CoV-2 spikes is ACE2 [17]. Through the six amino acids of RBD, SARS-Cov-2 binds to the ACE2 receptor with high affinity. Thus, it is possible that either i) SARS-CoV-2 passed from animal to human and adapted to binding ACE2 by interhuman propagation, following which it caused a pandemic, or ii) the ligand-receptor co-evolution took place in a non-human host [9].

In the lung tissue, ACE2 expression is relatively low, indicating the likelihood that the virus will use other co-receptors to promote a significant infection of cells. Candidate molecules for these co-receptors, which present a tissue distribution pattern similar to that of ACE2, are peptidases, including alanyl-aminopeptidase (ANPEP), DPP4, and glutamyl amino-peptidase (ENPEP), which are already recognized as receptors for the human coronaviruses [18]. Another co-receptor associated to ACE2 is the transmembrane serin protease 2 (TMPRSS2).

One of the other potential receptors promoting the transmission of SARS-CoV-2 is the neutral aminopeptidase (APN), a surface enzymatic glycoprotein abundantly expressed in a wide range of tissues, including endothelial, epithelial, fibroblasts, leukocytes, brain, respiratory tract [19], and renal tubules [20]. APN is a Zn-dependent protease (metalloprotease) that removes the amino acid sequence from the N-terminus of oligopeptides in the small intestine, but also plays a role in processing peptides and generating antigenic epitopes presented in association with the major histocompatibility complex (MHC class II) molecules. Both ACE2 and APN are components of the renin-angiotensin system (RAS). It has been proven that the presence of APN is not sufficient to produce the infection of the respiratory tract in pigs [21], suggesting that there might be factors other than receptors playing a role in CoV tropism, such as cellular proteases, which are activating the spikes [22]. Among the viral S glycoprotein, the hemagglutinin esterase (HE) of the viral peplos allows interactions with sialic acid residues on host cell surface and may enhance the ability to invade host epithelial cells of the respiratory tract [23]. The HE of β-coronaviruses HCoV-OC43 and HKU1 shares 30% homology with the HE of the influenza virus type C. SARS-CoV-2 can use the same receptors to infect different animal hosts, making proteomics a useful tool to identify the intermediate hosts that allowed the transition of the bat virus to humans [17].

### 4.2. Multiplication Cycle 

**Figure 2:**
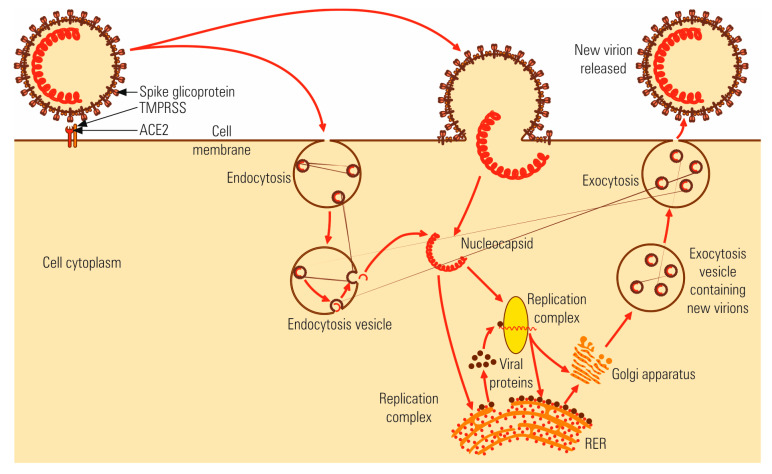
Schematic representation of coronaviruses multiplication cycle.

The multiplication cycle follows the stages characteristic for viruses with a genome of positive polarity [24]. According to the SARS-CoV model [25], the SARS-CoV-2 nucleocapsid is released into the cytosol after the fusion of peplos with the cytoplasmic membrane (Figure 2). The fusion of peplos with the cytoplasmic membrane (both possessing a lipid bilayer structure) is mediated by proteins embedded in the viral peplos [26]. After binding to cellular receptors, the virion is endocytosed into the endosome. Acidification of the endosome under the action of H+ pumps induces a conformational change in protein S, which is cleaved by furin at S1/S2 site and S2′ site. Importantly, the phylogenetic analysis of SARS-CoV-2 revealed the presence of an insertion of four essential amino acids (Arg-Arg-Ala-Arg). at the S1/S2 site of SARS-CoV-2. This unique furin-like cleavage site in the spike protein is missing in other coronaviruses. It is believed that this is a gain-of-function insertion, makes the entry of SARS-CoV-2 into cells easier, hence speeding up viral transmission [27]. The presence of furin in the lungs, liver, and the small bowel explains the tropism of the virus for these tissues, as well as the digestive symptoms observed in patients infected with SARS-CoV-2 (liver failure or diarrhea) [28]. Coronaviruses are the only enveloped viruses that infect the intestinal tract. Besides infecting small intestine enterocytes, SARS-CoV-2 is also resistant to the gastric acidity, as well as the detergent action of bile salts. After cleavage of the S protein, the viral peplos fuses with the cytoplasmic membrane in a sequence identical to the interaction of the HA influenza virus, with the epithelial cells of the respiratory tract. 

In coronaviruses, protein S mediates both receptor binding and fusion through the two domains, S1 and S2. In addition to the two endocytosis pathways, dependent on clathrin and caveolin, another independent pathway has been proposed, represented by the lipid pontoons rich in cholesterol and sphingolipids of the cytoplasmic membrane. Further penetration mechanisms of fusion (endocytosis, lipid pontoons) expand the spectrum of tissue cells in which the virus can multiply [25].

The ss RNA genome of SARS-CoV-2 consists of approximately 30,000 nucleotides and have a positive polarity (mRNA translated directly into proteins), being the largest of the known RNA viruses [29]. The genome has the following sequence: 5 ‘leader-UTR-Replicase-S (spike)-E (Envelope)-M (Matrix)-N (Nucleocapside)-polyA 3′. The leader sequence consists of approximately 75 nucleotides, and the UTR (untranslated region), consists of several hundred nucleotides. The genomic sequence, organized in at least 6 open reading frames (ORFs), is translated into 27 proteins, most of them, nonstructural (NS). The replicase gene, located at the 5′ end, occupies 2/3 of the genome sequence. The ORF 1a and ORF 1ab sequences are translated into polyproteins 1a and 1ab, respectively [30]. The two polypeptides encoded by the replicase sequence are cleaved by 2-3 virus-encoded proteases, but certain amino acid sequences are self-cleavage sites. The result is 15-16 NS proteins (NS 1-10 and NS 12-16), some of which assemble in the RNA polymerase (RNAP) complex, with a role in genome replication and transcription, with others demonstrating a regulatory role [31,32]. Some NS proteins act as virulence factors for SARS-CoV-1 and SARS-CoV-2, achieving IR blockade [4].

RNAP catalyzes the synthesis of a strand complementary to the genomic matrix sequence and results in double-stranded RNA, the structural support of replicative synthesis, and transcription of genetic information on the antigenomic strand.

At the 3′ end, the four significant structural genes encode the nucleocapsid protein (N), the S-spike protein, the E protein (deeply anchored in peplos), and the matrix protein (M) [33], which play a role in virion assembly.

The mRNA is transcribed by the RNA-pol complex, on the negative (antigenomic) polarity chain, in a set of seven molecular variants with subgenomic length sequence. All seven types of mRNAs with subgenomic length have the same 5′ leader sequence, – 75–78 nucleotides, derived from the 5′ end of the genomic RNA and the 3′ terminal sequence. The leader 5′ sequence probably remains associated with RNA-pol and is thus transcribed into each type of mRNA. Each mRNA molecule contains the entire sequence of the smaller molecule, and also has a different additional gene at the 5′ end. For most mRNAs, only the 5′ ORF is translated. Structural proteins S, M, and E are synthesized and inserted into endoplasmic reticulum (ER) membranes, following the secretory pathway to the Golgi complex. Protein N is synthesized by free polyribosomes and associates with genomic RNA molecules, forming a helical nucleocapsid [34].

Upon contact with the ER membranes and Golgi cisterns, the nucleocapsid is covered with peplos by budding. The glycoprotein spikes S, E, and M are included in the peplos. The peplos of this virus possesses extremely low concentration of cholesterol, unlike the enveloped viruses that are released by budding through the plasma membrane, explaining the pleomorphism of SARS-CoV-2. After assembly, virions are transported in vesicles to the cell surface and are released [34,35].

## 5. Genetic and Serologic Variability

Each coronavirus has a large number of serotypes, which do not give cross-serological reactions [36]. Thus, immunization against a specific serotype maintains susceptibility to infection with other serotypes of the same coronavirus.

Animal coronaviruses have a marked tendency for genomic variability, both by point mutation and by the more complex process of recombination [37].

The point (nucleic base substitutions) mutation rate of SARS-CoV is estimated at 0.80–2.38/10^3^ nucleotides/year. Probably, a similar rate of mutations occurs in the SARS-CoV-2 genome: transitions, with a rate of 1.67–4.67/10^3^ /site/year; transversions, with a rate of 1.16–3.30/10^3^/site/year.

The riboviruses mutation rate is dependent on the functional peculiarities of RNA-dependent RNA polymerase (RdRp). RdRp (also known as nsp12) is a main component of the replication/transcription machinery. SARS-CoV-2 shares high homology for nsp12 with SARS-CoV, implying that its function and mechanism of action might be well conserved [38]. In SARS-CoV, an exonuclease activity harboring proof-reading function has been reported for the nsp14 (ExoN), and a homologue nsp14 protein is also reported for SARS-CoV-2 and is involved in correcting nucleotide incorporation errors made by RdRp [39] thereby increasing the fidelity of RNA synthesis. The 3′–5′ exoribonuclease activity providing proof-reading function is a unique feature of CoV genomes among other RNA viruses because it enables slower mutation rates.

Genome sequencing of 220 viral strains showed a high incidence of mutations in the genomic region of replicase. Mutations guide the evolution of viruses regarding adaptation to multiplication in new cellular substrata, avoidance of defense mechanisms, or resistance to therapeutic agents [40]. 

Zoonotic transmission of SARS-CoV-2 appears to be associated with two point mutations with maximum nucleotide sequence variability: sequence 8789 and 28151 (Ser-Leu change) located in the polyprotein and ORF 8 genes, respectively [11].

Owing to the large size of the genome (27–31 kb), which makes it particularly prone to errors during RNA synthesis, coronaviruses have recombination processes with a much higher frequency compared to other riboviruses [41]. For a recombination event to occur, at least two compulsory conditions are required: the genomes of different viruses must be present in the same cell, through the phenomenon of co-infection or mixed infection; the genome of the two viruses have structural and nucleotide sequence homology [42]. Recombination can occur between two viral strains of the *Coronaviridae* family, but also with an unrelated virus. Recombination events, as well as events associated with the reassortment of genomic segments to *influenza A* viruses, generate a new virus, which, if transmitted to humans, may have a very high pathogenic potential [43]. In nature, such recombination (in *Coronaviridae*) or re-assortment of genomic segments (in influenza A) processes have an unknown frequency, but some of them generate new viruses with an increased capacity to disseminate in new hosts. There are several types of recombination found in RNA viruses.

RNA-RNA recombination involves the exchange of genetic information between genomic RNA unit molecules. Homologous recombination involves the reciprocal exchange of sequences between two similar or closely related RNA molecules with extended homologous nucleotide sequences. Thus, recombination can generate various genomic RNA molecules, from which functional RNA is selected [44].

Non-homologous recombination occurs between RNA molecules without sequence homology. The crossing-over sites of the two RNA molecules may have similar secondary structures [29]. In coronaviruses, non-homologous recombination is suggested by the HE spike, whose amino acid sequence is 30% homologous to the HE of the influenza C virus.

The mechanism of RNA-RNA recombination is most likely the copy-choice one. This phenomenon occurs due to the functionality of RNAP: the enzyme must pass from one template to another during RNA synthesis, to dissociate from the original template, and switch to a new proximal template, which can be cellular mRNA. It appears that the polybasic cleavage site of S-cleavage under the furin activity (Arg-Arg-Ala-Arg), flanked by three carbohydrate residues) of SARS-CoV-2 occurred due to the insertion of 12 nucleotides or a recombination process. The origin of the 12 nucleotides could be in the cellular mRNA. The insertion of proline presumably results in the inclusion of the three carbohydrate groups that flank the polybasic site [9].

## 6. COVID-19 Pathology

SARS-CoV-2 infects the cells of the oropharyngeal epithelium and upper respiratory tract. Based on epidemiological investigations, the average incubation period of COVID-19 is considered 3 to 7 days. However, it can vary from 0 to 24 days, depending on the amount of virus and immune reactivity. The vast majority of respiratory viral infections result from contamination with a small number of viral particles and are subclinical or are accompanied by mild to moderate flu-like symptoms [45]. Fever, fatigue, and dry cough are considered the main clinical manifestations, but less often, other symptoms may occur, including nasal congestion, rhinorrhea, pharyngeal pain, myalgia, and diarrhea. Asymptomatic or relatively mild infections develop more frequently in children: an opinion considers that the children keep a better-preserved immunological memory acquired after common cold coronaviruses infection; other authors consider that BCG or measles attenuated vaccines stimulate the cellular innate immune response. In vulnerable individuals (underlying pathology, immunosuppressed, elderly), the incubation period is short, and 15% of those confirmed positive progress to severe form [46].

The SARS-CoV-2 infection occurs in 3 stages: (1) incubation period, asymptomatic, with or without virus detectable by genomic test; (2) the period with mild symptoms, in which the virus is always detectable; (3) the period with severe respiratory symptoms and high viral load. 

In severe forms of SARS, the infectious process progresses from the upper respiratory tract into the epithelial cells of the bronchial tree. Especially in individuals with underlying pathologies, SARS-CoV2 infects pulmonary alveolar epithelial cells via RBD to ACE2. Alveoli are made up of 2 types of epithelial cells: type I (squamous) and type II pneumocytes, which release a surfactant, a mixture of phospholipids that reduces the surface tension of water molecules and prevents alveolar collapse. Type II alveoli cells are involved in repairing lung damage. The infection of these cells, harboring a high density of ACE2 receptors, blocks the regenerative response of the lung and aggravates respiratory difficulties [47]. Moreover, viral multiplication led to the cellular lysis, so that the cytoplasmic content is released and amplifies the inflammatory response and pyroptosis. Alveolar macrophages protrude into the alveoli, while others are free in the alveolar space. 

In severe cases, dyspnea and hypoxemia (pneumonia) occur frequently in the second or third week after infection. In critical cases, the pathological process can progress rapidly, with the onset of acute respiratory distress syndrome, septic shock, difficult to rebalance acidosis, hemorrhage and coagulation dysfunctions, thrombocytopenia, tachycardia, multiple vital organ failure characterized by hyperbilirubinemia, decreased diuresis, and impaired cognitive function [48]. Patients with severe or critical forms may present a moderate fever or even normal temperature. The elderly and those with chronic diseases generally have a poor prognosis. Clear signs of viral pneumonia include low levels of partial O_2_ blood pressure (<92%) and a characteristic radiological picture. In the initial stages of COVID-19 infection, the pulmonary radiological picture indicates areas of infiltrative opacification, located peripherally and postero-basally. As the pathology progresses, the infiltrates (with monocytes/macrophages) expand in both lungs, with pleural effusion and consolidations appearing as “opaque glass”. Additionally, lymphopenia (especially of NK cells), leukopenia, and increased levels of inflammatory markers (C-reactive protein, ESR, and pro-inflammatory cytokines - IL-6, TNF-α, IL-8) are recorded, but with normal procalcitonin values [49].

Radiological examinations reveal multiple small patched shadows and interstitial changes, especially in the lung periphery. As the disease progresses, patients show multiple ground glass shadows and infiltration shadows in both lungs. In severe cases, lung consolidation may occur. Pleural effusion is seldom observed in patients with COVID-19 [50].

The fluid flooding the alveolar space contains hyaluronic acid, the synthesis rate of which is extremely high in the lungs. Elevated levels of pro-inflammatory cytokines (IL-1, IL-6 and TNF) stimulate hyaluronan synthetase 2 activity in endothelial cells and fibroblasts. The liquid component exuded in the alveoli is viscous and does not return to the blood as hyaluronate is hydrated with a considerable amount of water, 1000 times higher than its molecular weight. Therefore, hyaluronidase is considered a therapeutic option for the elimination of viscous lung exudate [46].

The SARS-CoV-2 infection induces severe immune system (IS) dysfunctions, including spleen and lymph node atrophy, functional depletion of T lymphocytes, manifested by a decreased number of Th and Ts lymphocytes in secondary lymphoid organs, especially in critically ill patients. The percentage of naive Th lymphocytes increases, while memory Th and T regulatory lymphocytes decrease [50]. In some patients, marginally elevated levels of liver enzymes, lactose dehydrogenase (LDH), muscle enzymes, and myoglobin may be observed. In critically ill patients, high levels of troponin and D-dimers, resulting from the degradation of fibrin clot under the influence of plasmin, can be detected. Additionally, vasculitis (endothelial lesions, congestion of the alveolar septal vessels, thickening of the small vessel wall, lumen stenosis, and occlusion), hypercoagulation with hypoxic gangrene of the extremities, and multi-organ lesions (lungs, heart, liver, and kidneys) characterized by local hemorrhagic necrosis have been reported [50].

It is obvious that from the clinical point of view, we are facing a complex disease, with upper and lower respiratory, as well as digestive symptoms. While some people develop respiratory symptoms, others exhibit digestive symptoms and the reason behind this is still unknown. It was seen that in children, most of the cases are digestive, and, according to the Canadian Society of Pediatricians, this might be related to the distribution of ACE 2 receptors. Another interesting fact is that a lot of youngsters present only with hyposmia, but they are not aware of it. Dysgeusia/hypogeusia/ageusia (taste sense distortion reduction/complete loss) have also been reported in patients with COVID-19 infection [51]. The sense of smell is progressively affected (this is also encountered in flu infection for example) while the patient unknowingly spreads the infection until loss of taste also appears. This shows a potential of virus to infect specifically neurons of the olfactive nerve, without other symptoms of upper respiratory tract. It is well known that viruses affecting respiratory tract can cause loss of smell after infection, and there are over 200 viruses causing respiratory infection, from which previously known coronaviruses are 15% 

Here, it comes into question whether we can limit the spread of infection in this paucisymptomatic phase of infection. The main reason for which people with hyposmia are dangerous for the community is because they are not instructed to look for small symptoms like how they feel the smell of coffee, food, smoke, etc. An intensive campaign of education about possible symptoms could be effective and may be used as a screening tool for selecting infected individuals.

So, the way each individual reacts to SARS Cov 2 infection is still unclear. During lockdown periods, some hospitals were running PCR tests for screening at the time of admission for surgeries. A number of patients were detected positive. Our personal remark was that patients with cancer (an immunosuppressive disease) were completely asymptomatic for COVID 19. 

### 6.1. Immune Response to SARS-CoV-2 Infection

In the first two stages of clinical infections (incubation and mild symptoms), the cell-mediated immunity (CMI) and humoral factors play a crucial role in modulating the host response to viral infection.

Early administration of IFN-I ameliorates the pathological response to inflammation [52]. It seems that in clinical cases or in experimentally infected mice receiving high infection dose, the viral infection of alveolar cells, macrophages, and interstitial CD is not followed by the IFN-I synthesis [53]. The delay in the IFN-I response functions as a pro-inflammatory signal, amplifying the massive influx of neutrophils and monocytes-macrophages, responsible of the typical pathological picture of SARS infection, necrosis of pneumocytes and alveolar macrophages, production of alveolar vascular exudate, respiratory failure, and the fatal outcome [45].

The effectors of innate CMI are non-specific cytotoxic cells, activated within minutes after recognizing the viral antigenic structures on the surface of infected macrophages or dendritic cells (DC), natural killer (NK), K cells, Tγδ lymphocytes, and neutrophils. The long incubation time is directly correlated with the anti-viral protective role of macrophages, dendritic cells (DC), NK, and K cells, as well as neutrophils. Neutrophils and macrophages internalize any released virions and neutralize them while NK and K cells detect infected cells by unknown molecular mechanisms and lyse them [46]. 

The physiological state of macrophages influences in a decisive way the clinical evolution of infection from inapparent to mild or severe. Resident tissue macrophages recognize a well-preserved set of pathogen-associated molecular pattern antigens (PAMPs) via toll-like receptors (TLRs) 1–10 [54]. From these, TLR 7 recognizes ssRNA [55]. Unlike TLR, NOD (nucleotide oligomerization domain) -like receptors (NLR) and Rig (retinoic acid-inducible gene)-like helicase (RLH) receptors consist of soluble cytoplasmic proteins that monitor this compartment for intracellular invader signals and are specialized to receive PAMP and D(danger)AMP- signals. Two helicases, RIG-1 and MDA5, act as cytoplasmic sensors of viral RNA. After stimulation of the two helicases, NF-κB and IRF3/7 are activated, and gene transcription for IFN is induced [56].

At this stage, the influx of neutrophils, which always accompany the host invasion by any infectious agent, is moderate. Viral multiplication is blocked or occurs at a low rate, which could explain the negative genomic test.

SARS-CoV-2 has special mechanisms that inhibit the macrophages, especially those impeded by pollutant factors (like smoke). The virus suppresses early pro-inflammatory response mediated by IFN-I and effector inflammatory cytokines (IL-1, IL-6, TNF-α). Inside the cell, virion remains untouched, and multiplication cycle continues. Monocytes/macrophages have a lower density of ACE2 receptors, making less probable a direct infection, but immune complexes formed by virions and anti-SARS-CoV-2 antibodies bind to Fcγ receptors and are engulfed, resulting in the internalization of viral particles and subsequent infection (antibody directed enhancement) [23].

**Figure 3:**
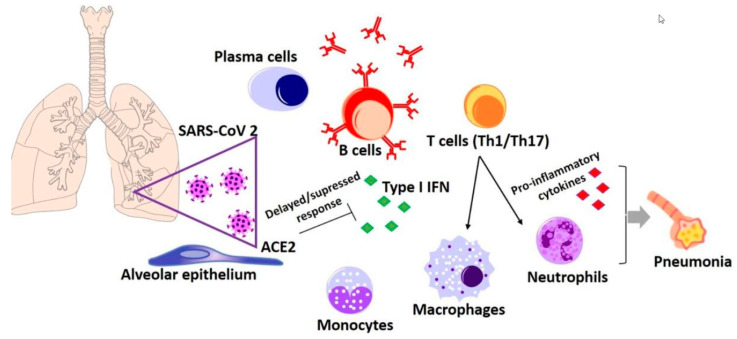
Schematic representation of the CMI effectors in SARS-CoV 2 infection.

Specific immune cells are represented by TCD4 and TCD8 (cytotoxic) lymphocytes [57] (Figure 3). Activation of the specific CMI provides long-term protective memory as it is geared towards highly conserved internal viral proteins. The efficacy of the primary CMI response in COVID-19 depends firstly on the reactivity of TCD8 lymphocytes [52,58]. If the CMI response is inadequate, the SARS-CoV evade immune recognition, the infectious process enters the phase of severe clinical manifestations, and the virus multiplies in the epithelial cells of the deep bronchial tree, producing extensive lesions in sensitive cells, especially those positive for ACE2: lung alveoli cells, enterocytes, and renal epithelial cells [46].

Humoral mediated immunity (HMI) memory, assessed after SARS-CoV infection, is short due to the emergence of new mutational antigenic variants [59]. 

Severe manifestations of the infection occur by the excessive activation of the inflammatory process. Infected epithelial cells stimulate inflammation in the lung tissue, resulting in a significant influx of neutrophils and macrophages. The infection becomes viremic, and the inflammatory reaction becomes systemic, with the involvement of the digestive tract, kidney, liver, and heart muscle, thus endangering life. From a molecular standpoint, the severe clinical stage is characterized by the “cytokine storm”: neutrophils, eosinophils, macrophages, and DCs migrate into the infectious foci, and release an entire cascade of pro-inflammatory ILs. IL-1, the primary mediator of the inflammatory reaction, released by neutrophils and macrophages through the autocrine loop, stimulates the synthesis of IL-1, IFNγ, and tumor necrosis factor (TNF) in macrophages [60], which further induce the synthesis of IL-2, -4, -7, -10, -12, and -13, which, on their turn, are chemoattractants for monocytes (MCP) and macrophages (MIP). IL-1 stimulates the synthesis of IL-6, which induces the hepatic synthesis of acute-phase proteins, chemokines, and C-lectins (e.g., mannose-binding lectin). This systemic hyperinflammatory process could lead to the occurrence of lesions, especially in lungs, but also in the liver, kidney, and heart. In the lungs, monocytes/macrophages massively infiltrate the pulmonary interstitium and impede the alveolar gas exchange. A significant number of patients develop cardiovascular morbidity with troponin rise, tachyarrhythmias, and thromboembolic events [23].

The number of TCD8 lymphocytes and NK cells decreases by approximately 50%, but the remaining cells secrete perforins and other cytotoxic factors with lesional effects [61].

Complement is activated in the acute inflammatory foci, and the resulting anaphylatoxins, C3a and C5a, amplify the pathological process of the acute respiratory syndrome [48]. C5a plays a critical role in the pathogenesis of SARS-CoV. First, it interacts with neutrophils via C5aR, diminishes their function to paralysis, and inhibits their apoptotic death. The prolonged viability of neutrophils amplifies the inflammatory process, lymphatic transvasation, and respiratory failure, leading to hypoxia and cardiac dysfunction, decreased blood pressure, and blood stasis due to thrombus formation. These changes induce functional insufficiency in the major organs (heart, liver, kidneys, and lungs), with fatal outcome [62]. Additionally, C5a induces the synthesis of IL-8, which stimulates fibrin clot formation, activates thrombogenesis and endothelial reorganization. The anticoagulants are thus a key component of the therapeutic schemes in septic shock pathology [48,63].

**Figure 4:**
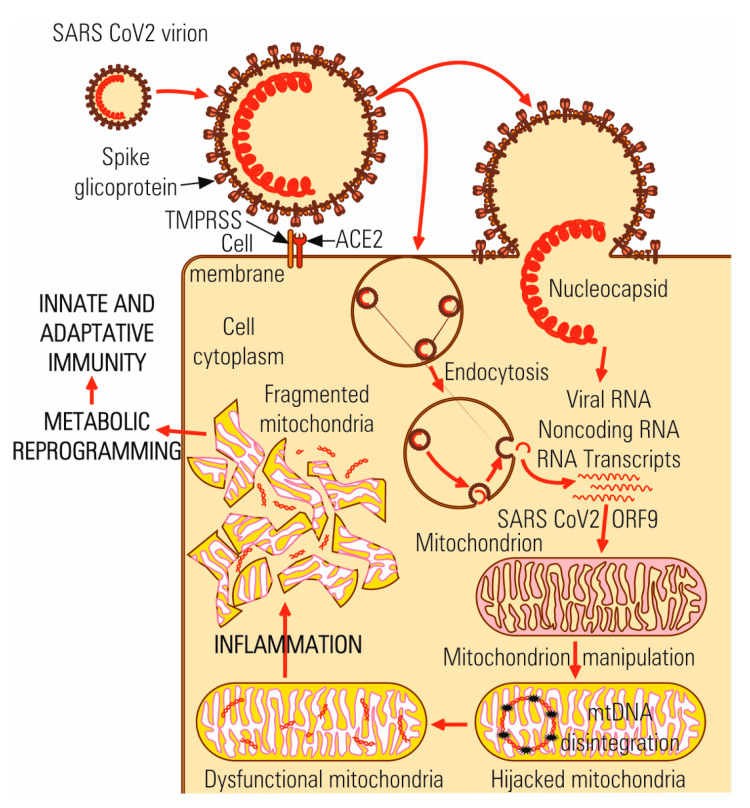
Hijacking mitochondrial metabolic processes in Covid-19 infection.

Recent studies suggest a central role of mitochondria in the host answer to viral infection and immunity. Mitochondrial external membrane is harboring the DAMP receptors [64,65,66,67] involved in inflammation [68] (Figure 4). The viral proteins interact with mitochondrial proteins which are involved in energy metabolism, that leads to immune anti-viral response suppression [69].

Chen et al. (2019) agreed that there are physiological mitochondrial differences between sexes, which may partially explain gender differences in Covid-19 patients’ pathology and mortality [70]. The mitochondria function is regulated by sex hormones. The expression of TMPRSS 2 co-receptor is induced by androgens and is regulated by specific receptors localized in the mitochondrial compartment. Higher prevalence mortality in men is partially owing to the higher TMPRSS 2 expression induced by androgens. In silico analysis showed that genomic and all subgenomic ARN molecules had a greater density în mitochondria and nucleolus. However, the transport mechanism of SARS-CoV-2 RNA in mitochondria remains unknown [71]. The genetic polymorphism of ACE2 gene identified in different human populations seems also to influence the mitochondria function. Patients with comorbidities or with a special ACE2 polymorphism, which are more susceptible to SARS-CoV-2 infection also exhibit mitochondrial disfunctions. The exonic variant—G8790A is significantly more frequent in European and South Asian populations [72], while the G/G genotype may decrease ACE2 expression on the cell surface up to 50% [43,73].

### 6.2. Inflammation-Triggering Risk Factors in COVID-19 Infection

The persistent inflammatory condition associated with age, metabolic syndrome, and obesity seems to partially explain the higher susceptibility of this population groups to COVID-19.

Age is one of the risk factors for COVID-19. Among the multiple physiological changes occurred in the elderly, some pare predisposing them to COVID-19, i.e., immunosuppression and immunosenescence (senescent cells are acquiring a specific phenotype called SAPS = senescence-associated secretory phenotype, that maintains an inflammatory condition; oxidative damage of all types of macromolecules; a prothrombotic state, manifested by increased plasma levels of fibrinogen and IL-6; increase in plasma levels of TNF-α and CRP, even in the absence of an evident inflammatory process [74,75,76,77,78]. The low level of inflammation associated with the aging process was termed *inflammaging* [77].

Inflammation invariably accompanies different infections (bacterial, fungal, parasitic and viral), but is also triggered by noninfectious inflammatory processes such as rheumatoid arthritis (RA), autoimmune diseases, and neoplasms] [79,80]. As an anti-infective response, lipopolysaccharide (LPS)-stimulated macrophages secrete lipocalin 2, a protein that binds bacterial siderophores and sequestrate Fe. A persistent inflammatory condition associated with obesity, aging, and kidney failure creates the conditions for anemia.

Moreover, vitamin D deficiency is associated with inflammatory processes. Vitamin D and its synthetic analogs act as an anti-inflammatory and, consequently, as stimulators of innate and adaptive immune reactivity. Innate and adaptive immune cells have vitamin D receptors. Receptor activation after vitamin D binding stimulates the IL-10 anti-inflammatory cytokine synthesis by T lymphocytes.

The rate of SARS-CoV-2 infections, as well as the mortality rate, seem to be higher among men. Women are less sensitive owing to the innate immune system, and specific factors (sex hormones) that provide better anti-infective protection. Inflammaging level is different in men and women, with a higher IL-6 level in men.

After viral infection, mitochondrial DNA is released in extracellular space and function as a DAMP signal (danger-associated molecular patterns), inducing the systemic inflammatory and innate immune response. Mitochondrial DNA release is an old mechanism of response to stress, well conserved in evolution and is induced after infection with many types of RNA and DNA viruses. In aged patients with Covid-19 infection are reported high levels of mitochondrial DNA [81].

Another factor which may amplify morbidity of aged patients is the differential ACE2 expression on the cells of different tissues, but potentially also between individuals (men/women, children/adults). Some studies suggest that ACE2 expression is highest in children and young women and decreases with age [82], being the lowest in individuals with chronic diseases, including type II diabetes, hypertension, experiencing severe consequences of Covid-19 infection. In contrast, ACE2 is overexpressed in women, comparatively to men [70].

Diabetes and hypertension are comorbidities that increase the risk of SARS-CoV-2 infection [83]. In patients with type 1 and 2 diabetes, the density of ACE2 increases significantly in the epithelial cells of the lung, kidney, small intestine, and vascular endothelium. Treatment includes ACE1 inhibitors and type I receptor blockers for Ang II. Patients with hypertension are treated in a similar manner utilizing ACE inhibitors. The inhibition of the Ang II pathway leads to the expression of ACE2 on the membrane of concerned tissue cells. Additionally, ibuprofen and thiazolidinedione have demonstrated an identical effect. Although ACE2 expression facilitates SARS-CoV-2 infection, its recombinant form has been suggested for the treatment of inflammatory lung disease, cancer, diabetes, and hypertension. An increased polymorphism of ACE2 was associated with the pathology of diabetes, hypertension, or stroke, which could also impact the evolution of the infectious process produced by SARS-CoV-2 [84]. Diabetes is not only a significant risk factor for the severe course of the SARS-CoV-2 infection, but the virus directly binds to the ACE2 receptors from the pancreatic islet cells, causing their lysis [85]. Furthermore, immune-mediated inflammatory diseases (IMID) (RA, ankylosing spondylitis, Crohn’s disease, ulcerative colitis, psoriasis, and atopic dermatitis) are characterized by infiltration of the target tissue with granulocytes and macrophages. Therapies for IMID are aimed to inhibit the pro-inflammatory cytokines. RA responds to TNF and IL-17A inhibitors; Crohn’s disease, ulcerative colitis, psoriasis, and ankylosing spondylitis respond to TNF and IL-23 inhibitors; atopic dermatitis responds to IL-4 and IL-13 inhibitors [86].

In smokers, pulmonary macrophages, including alveolar macrophages, are activated by the irritating effect of tars, secreting pro-inflammatory ILs (IL-1, TNF, IL-6), with positive chemotactic action toward neutrophils, which infiltrate the lung parenchyma. The local immune response is repressed by an excess of free radicals. Smokers require four times the vitamin C/day to counteract the oxidative effects of free radicals. Following macrophage activation, the inflammatory process is amplified and turns chronic [87].

#### Involvement of the Renin-Angiotensin System (RAS) in the Pulmonary Pathology of SARS-CoV-2

The acute respiratory syndrome is mediated by the RAS system, which plays an essential role in controlling the cardiovascular and renal function, by maintaining the blood pressure homeostasis and the hydroelectrolytic balance. In RAS, various components of the system are synthesized and result from a large precursor—angiotensinogen (synthesized in the liver) into a complex system of enzymatic activities. The activity of this system is initiated by renin, a serine protease synthesized by the cells of the renal juxtaglomerular complex, which converts angiotensinogen to angiotensin I (Ang I). Ang I, the major effector of the RAS, is cleaved by ACE (angiotensin-converting enzyme) to Ang II, in two stages: the first stage results in peptide 1–9, probably inactive, later undergoing cleavage of 2 amino acid residues at the C-terminus, and resulting in the active peptide 1–7 [85,88]. The conversion of Ang I to Ang II can be also be mediated by chymase, a serine protease.

Ang II binds to specific type 1 receptors (AT1R) on vascular endothelial cells and, by prolonged action, in the absence of a specific inhibitor, induces several effects: pro-inflammatory activities, vasoconstriction, hypertension, and hypokalemia, and activates the secretion of antidiuretic hormone and aldosterone, pulmonary fibrosis, and proliferation of cells in the inflammatory foci.

ACE2 facilitates SARS-CoV-2 entry into cells, and the presence of ACE2 as a virus receptor on different types of cells probably amplifies the disease pathogenesis, which manifests itself as a multi-organ systemic disease, with pulmonary, but also of the intestinal tract, liver, and kidneys involvement [61]. ACE2 also plays a very important role in the control of the inflammation, and therefore, in the evolution of pathological process. ACE2 binds to specific AT2R (angiotensine-2 receptor) receptors and counteracts the pro-inflammatory effects of Ang II. ACE2 counteracts vasoconstriction, modulate leukocyte migration, prevents the occurrence of acute pulmonary insufficiency. Both in SARS-CoV-1 and SARS-CoV-2 infections, ACE2 levels decrease because the virus uses this enzyme as a receptor. The deficit induced by the infectious process of ACE2 increases the level of Ang II and disrupts cardiac contractility [88].

## 7. SARS-CoV-2 Diagnosis

The samples collected for the diagnosis of SARS-CoV-2 are from the respiratory tract, including nasopharyngeal and oropharyngeal swabs, bronchoalveolar lavage, tracheal aspirate or sputum, as well as urine, blood, stool, autopsy material, and lung tissue [89]. The diagnostic tools most widely used at the moment are summarized in Table 1.

Globally, the most widely used test to confirm the diagnosis of COVID-19 is reverse transcription-polymerase chain reaction (RT-PCR). Furthermore, several types of tests have been developed worldwide to combat the spread of SARS-CoV-2. In Germany, a highly specific RNA RT-PCR technology has been developed that does not interfere with other coronaviruses. This assay detects SARS-CoV-2 RNA in the viral envelope and uses an RdRp [90]. Another diagnostic approach involves the detection of ORF1b and N regions in the SARS-CoV-2 structure, in approximately 75 min. However, the regions considered are highly conserved in several coronaviruses, and hence, the degree of specificity for SARS-CoV-2 decreases [91]. Another approach involves the detection of genes encoding RdRp and helicase specific for SARS-CoV-2. Among specificity, another advantage of this approach is the high sensitivity, that allows the test to be used for samples with low viral loads [92]. Other globally recognized RT-PCR tests include the Xpert^®^ Xpress SARS-CoV-2 test and the Vivalytic COVID-19 test. The Xpert^®^ Xpress SARS-CoV-2 test was developed in the USA and allows the detection of SARS-CoV-2 in 45 min, in various specimens such as nasopharyngeal swabs, nasal washings, or other aspirates [93]. The test was approved by the Food and Drug Administration (FDA). The Vivalytic COVID-19 test was developed in Germany and can detect SARS-CoV-2 within approximately 2.5 h [94] (https://www.bosch.com/stories/vivalytic-rapid-test-for-covid-19/). Another molecular detection test for SARS-CoV-2 is Abbott ID Now ™ COVID-19, utilizing isothermal amplification technology of nucleic acids for the qualitative detection of viral RNA in 5 min. Owing to the use of a portable instrument, it can be used at any location, without necessitating specialized laboratories. The identification of SARS-CoV-2 is based on the detection of the gene encoding RdRp in various samples, including nasal, nasopharyngeal, and oropharyngeal swabs [95] (Abott-site, 2020).

In addition to molecular diagnostic tests, several serological tests are available for the detection of SARS-CoV-2 based on viral proteins and antibodies present in the blood and plasma. The most widely used biomarkers for the detection of SARS-CoV-2 are IgG and IgM antibodies, which can be detected in infected patients approximately two weeks after infection. IgM can be observed in patients within 10–30 days of infection onset, while IgG can be detected 20 days after infection [96]. Several companies have developed tests that allow the detection of synthesized IgM and IgG antibodies in response to the SARS-CoV-2 infection. One known rapid test is performed by BioMedomics in the USA, with the ability to detect IgG and IgM antibodies in about 10 min, starting with a minimum volume of blood or approximately 10 μL serum/plasma [97] (https:// www. biomedomics.com/products/infectious-disease/covid-19-rt). One of the most advanced rapid tests that has received FDA approval is the DPP COVID-19 IgM/IgG, developed by Chembio Diagnostics in the USA. The blood volume required is minimal, and the result is ready in approximately 15 min. Although there are many such tests available worldwide, further information is needed regarding their accuracy when compared to RT-PCR as a diagnostic test. In Europe, numerous reports have shown that rapid tests developed in China present an error rate of over 70%. Furthermore, IgG and IgM antibodies can be identified in patients two weeks after infection, and hence, biomarkers are needed to allow early identification of SARS-CoV-2.

Biochemical tests can provide additional information regarding the SARS-CoV-2 infection. Abnormal biochemical parameters were confirmed in patients with COVID-19 disease, the most common being leukocytosis, leukopenia, lymphopenia, elevated levels of CRP and lactate dehydrogenase, high erythrocyte sedimentation rate (ESR), and abnormal levels of liver enzymes [98]. In SARS-CoV-2, lymphopenia is a common condition, positively correlated with disease severity. Lymphopenia can be caused by a COVID-19-mediated “cytokine storm” or by direct action of SARS-CoV-2 [49]. Additionally, the inflammatory response triggered by the virus causes changes in PCR and ESR values in the blood.

Achieving an accurate result is essential to prevent the spread of the SARS-CoV-2 infection. Currently, all efforts are directed towards the development of diagnostic tests, as specific and accurate as possible. Although specialized laboratories, trained staff, increased constraints, and longer waiting periods are required, RT-PCR remains the most advantageous for confirming the COVID-19 diagnosis. Rapid tests that involve the detection of IgG and IgM antibodies can provide results within minutes, responding to the need to test as many individuals as feasible in the shortest time. However, further evaluations of these tests are required to improve result accuracy. Both biochemical and antibody (Ab) detection tests can be used in addition to RT-PCR diagnosis to increase the quality of results obtained and to identify additional information regarding the immune status of patients.

## 8. Pharmacology

Currently, no drugs with specific actions against COVID-19 are available. The currently available therapeutic approaches are summarized in Table 2. During severe manifestations, the therapy aims to reduce the side effects caused by infection, including inflammation, fibrosis, electrolyte imbalance, and support respiratory function [46]. A significant goal for patient treatment is IL-1 [56]. Commonly used anti-inflammatory drugs include NSAIDs, glucocorticoids, chloroquine/hydroxychloroquine, immunosuppressants, pro-inflammatory cytokine antagonists (IL-6R), monoclonal antibodies (MAB), TNF inhibitors, IL-1 antagonists, and Janus kinase inhibitors. Glucocorticoids are administered in small, short-term doses, only to patients in whom fluids and vasopressor therapy have failed to restore hydrodynamic balance [63]. Anti-inflammatory treatment is immunosuppressive, requiring a balance between benefits and risks. Anti-IL-6 MAB and anti-TNF MAB neutralize the pro-inflammatory action of the two ILs.

The viral infectious process is initiated by the interaction, sometimes with a high specificity of the viral ligand, with cellular receptors. As stated above, SARS-CoV-2 binds with a high affinity to ACE2, as well as to other receptors (e.g., neutral aminopeptidase). Soluble recombinant ACE2 can be used as a blocker of virus interaction with membrane ACE2.

Specific molecules (cyclodextrins, sterols) can reduce the coronavirus infectivity by inhibiting interactions with the cellular receptors, thus, interfering with the fusion of peplos and the cytoplasmic membrane of the host cell [11,118].

Regulatory kinase inhibitors of endocytosis block the entrance of the virus into cells. Baricitinib is an inhibitor of aminopeptidase 2(AP2)-associated protein kinase 1 (AAK1) and Janus family tyrosine kinases (JAK). In silico analyses indicate that drugs that inhibit AAK1 associated with neutral AP2 could inhibit target cell infection by endocytosis [119].

Some drugs may inhibit clathrin-mediated endocytosis of COVID-19. The endocytosis pathway, an alternative to fusion, involves endosomes and lysosomes. Inhibition of this pathway could present a potential target for the development of a novel therapeutic strategy, along with chlorpromazine or CQ (an antimalarial drug, whose mechanism of action involves alkalizing the endosome and inhibiting the activity of lysosomal enzymes) [120]. Additionally, NAK kinase inhibitors (AAK1 and GAK), baricitinib, used in the treatment of rheumatoid arthritis and an inhibitor of AAK1 and Janus kinase (JAK-STAT-kinase), apparently inhibits viral multiplication [26,121].

Remdesivir, an adenosine analogue that acts as an inhibitor of RNA-dependent viral RNAP, is useful for the treatment of infection symptoms [122]. However, exonucleases proof-reading can cleave phosphorylated Remdesivir from the RNA chain and lead to resistance [23]. In China, multicenter studies have shown the effectiveness of Remdesivir, a drug traditionally used to treat malaria (chloroquine phosphate), and introduced into the recommended treatment regimens for COVID-19 (500 mg 2 times a day, ≤ten days) [123]. By increasing endosomal pH, chloroquine (CQ), an acidotropic amine of quinine, can block the activity of lysosomal enzymes of macrophages, and thus inhibit the dissociation of N protein from genomic RNA. CQ inhibits the activation of active protein kinase p38 (MAPK), which is involved in the multiplication of HCoV-229E [120]. Hydroxychloroquine (HCQ) (hydroxylated chloroquine, used in the antimalarial treatment) is administered orally, demonstrating rapid absorption, and inhibiting prostaglandin synthesis (attracting neutrophils), IL-1 synthesis, and O_2_- release in monocytes. HCQ is concentrated in the lung, eyeball, and liver tissues, converted to the active (ethylated) form [124]. CQ and HCQ have immunomodulatory and anti-inflammatory effects; however, therapeutic benefits are attributed to the broad-spectrum anti-viral effect against viruses such as HIV, Marburg, Zika, Dengue, SARS-CoV1 [125]. CQ and HCQ could interfere with the virus binding to membrane receptors or the endosomal entrance through acidic pH-dependent endocytosis. Nevertheless, the effectiveness of HCQ in treating COVID-19 is highly controversial.

The original report proposing HCQ as a potential treatment for COVID-19 described a small cohort of 26 patients who were treated in an open-label, single-group study (200 mg/three times daily/10 days). Hydroxychloroquine was shown to be effective in reducing the viral burden in treated patients (65.0% clearance by day 5 compared to 18.8% clearance in untreated patients) [99]. A subsequent observational study by Geleris et al. performed on a cohort of 1376 COVID-19 patients showed that HCQ use was not linked to a significantly higher or lower risk of intubation or death [126].

The combination of antiretroviral agents lopinavir (protease inhibitor) and ritonavir, used in the treatment of SARS-CoV-1 infection, or the combination of remdesivir and lopinavir/ritonavir (also known as Kaletra and Aluvia) could present therapeutic options for COVID-19 [127]. Other treatment options include leronlimab, a humanized MAB (CCR5 antagonist) (ClinicalTrials.gov), and galidesivir, a nucleoside RNAP inhibitor [111].

Currently, clinical trials underway at ClinicalTrials.gov are investigating the efficacy of Remdesivir therapy, convalescent serum immunoglobulins, arbidol hydrochloride, inhibitor of viral peplos fusion with the host cell membrane, IFN-α nebulization, ASC09F + oseltamivir, lopinavir-ritonavir association, mesenchymal stem cell therapy, darunavir/cobicistat, hydroxychloroquine, methylprednisolone (glucocorticoid-induced immunosuppression may be delayed), other immunosuppressive drugs used in SARS therapy, pre/probiotic administration for preventing intestinal bacteria translocation and secondary infections, and plasmapheresis for critical cases with severe inflammatory reactions [128].

The widely accepted strategy for the management of COVID-19 is anti-viral, anti-shock, anti-hypoxia, anti-secondary infections and also aims to achieve hydro-electrolytic and acid-base host balance [129].

## 9. Prevention of SARS-CoV-2 Infection by Vaccination

The administration of a vaccine is based on a well-defined strategy. The purpose of vaccination may be to eradicate, eliminate, or limit an infectious process. Eradication refers to the disappearance of the pathogen following vaccination. Elimination corresponds to the disappearance of pathological manifestations, although the pathogen is preserved in the human or animal population. Limitation refers to the possibility of controlling an infectious disease to a level that is no longer considered a public health problem.

In the current SARS-CoV-2 pandemic, which has overwhelmed the world’s population lacking a specific immune memory, specialists are researching for solutions with different variants of a protective vaccine. Several vaccine formulations are currently under development and testing in clinical trials (Table 3). Vaccines can be prepared from pathogenic strains whose virulence has been attenuated by cultivation under particular environmental conditions, or from pathogens inactivated under the action of chemical agents such as formaldehyde, ethylene oxide, thiomersal, or β-propiolactone. In terms of complexity, vaccines may be unitary, containing all antigenic components of the infectious agent, or subunitary vaccines, prepared from fractionated antigenic components, which must compulsorily induce the synthesis of specific, neutralizing antibodies [130]. SARS-CoV-1, SARS-CoV-2, and MERS provide a cross-IR, which underlies the production of a broad-spectrum coronavirus vaccine [131].

Possible SARS-CoV-2 vaccine variants are inactivated vaccines, attenuated vaccines, recombinant S protein, and the S protein viral vector vaccine [132].

Given the high virulence of SARS-CoV-2, the SARS-CoV-2 candidate vaccine will be probably a chemically inactivated unitary or subunitary one, depending on several factors (production technology, costs, safety and efficiency). A promising candidate for a subunitary vaccine is the recombinant S protein or cDNA -encoding S protein [127]. The S-spike, prominent on the surface of the peplos anchors the virion to the membrane receptor (ACE2) of sensitive cells in the respiratory tract epithelium, oral mucosa, especially lingual mucosa. Another protective subunit vaccine could be the RBD of protein S, which recognizes the binding site of ACE2. The same RBD sequence could be used as a drug target inhibiting ACE2 binding [6,132]. The RBD of SARS-COV-2 has a greater affinity than SARS-CoV-1, explaining the higher contagiousness of this virus. Antibodies specific for the binding domain of the S protein to the cell receptor block the ability of both viruses (SARS-CoV-1 and 2) to fuse with the host cell membrane, thus presenting the potential to develop a vaccine with protection against both viruses [6].

However, the subunitary vaccine candidate will need to retain the antigenic determinants of the native molecule. The real risk of partial efficacy is combined with the minor risk of side effects induced by the lipid components of peplos, the inactivating agent, and the preservative. The preparation of the subunitary vaccine requires the technology to separate peplos glycoproteins by treatment with chemical agents. The risk of denaturation, loss of the native conformation of potentially immunogenic molecules, is even greater. Consequently, the probability of stimulating a significant humoral IR decrease.

The route of administration influences the intensity of the IR. The optimal routes of administration of inactivated vaccines, to which adjuvants are added to increase the persistence of the vaccine, are intramuscular (im) and subcutaneous (sc).

In SARS-CoV-2 infection, the protective effect of antibodies must be predominantly manifested in the epithelium of the respiratory and digestive tracts. An intramuscularly administered vaccine activates B lymphocyte clones, which, through the membrane immunoglobulin receptor, specifically recognize the vaccine epitopes. Stimulated lymphocytes proliferate, differentiate, and synthesize IgM, IgG, and, to a lesser extent, IgA antibodies. IgA is the only class of immunoglobulins that, in dimeric form, can be transferred to the surface of epithelia and could provide anti-SARS-CoV-2 protection at the entry gates into the host, suggesting that the application of topical products on the nasal mucosa or pharyngeal mucosa for the enhancement of the local resistance could contribute to the for limiting interhuman transmission.

The oral administration of the SARS-CoV-2 vaccine will probably be more useful for the protection of mucous membranes: first, the digestive tract, although to a lesser extent; secondly, the epithelial lining of the respiratory tract, due to the preferential recirculation of the lymphocytes in homologous structures (homing).

The strategy for obtaining an attenuated vaccine remains an alternative perspective. This method involves the selection of mutant strains of SARS-CoV-2 with attenuated virulence through successive passages on semi-permissive cellular substrates. The virus in the attenuated preparation, after subcutaneous or intramuscular administration, is transported by the blood and lymph, multiplying in the same tissues as the wild-type virus, without producing pathological manifestations, except, at most, in a mild form and ensuring full immunization. However, a major disadvantage of such a preparation is the risk of reversion by mutation to the virulent form.

The main advantages of attenuated viruses are the stimulation of the CMI response, which is essential for sterilizing the viral infection outbreak, as well as the lower production costs. However, there are several disadvantages with such vaccines, such as storage conditions at 4 °C until administration, the risk of spontaneous reversion to the virulent form, and administration to immunodeficient individuals may reproduce the pathological picture of the natural infection.

Among individuals positive for SARS-CoV-2, most have inapparent or mild infections. Some individuals may have maintained a residual state of immunity after a previous infection with a common cold coronavirus. This suggests the prospect of producing a vaccine after the model of E. Jenner’s smallpox vaccine, i.e., using an infectious coronavirus in animals, but harmless to humans, providing the biochemical and structural homology of the S spikes of the vaccine virus, with the S spikes of SARS-CoV-2 [133].

Given the chemical differences between viral and human proteins, inactivated and subunitary vaccines essentially stimulate humoral-mediated immunity.

SARS experience shows that specific antibodies persist for approximately 2–3 years after infection, and researchers expect a similar immune profile in SARS-CoV-2. Moreover, it has been observed that antibodies specific for the RBD of the S glycoprotein inhibit the virus’s ability to fuse with the host cell membrane and initiate the infectious process. Therefore, it is probable that the natural immunization of the population will result in the limitation of this pandemic.

The development of an effective and safe vaccine for human use is a long-term process (especially since there is currently no coronavirus vaccine on the market and no capacity for large-scale production of a potential new vaccine). In addition to this time horizon, further time is required for the large-scale distribution and administration of the vaccine, which can take several weeks. The vaccine will probably be available later than necessary to stop the first wave of the pandemic. However, it will undoubtedly help reduce mortality and morbidity in the scenario that the virus is maintained in the human population. The most advanced SARS-CoV-2 vaccine is in Phase I clinical trial (ClinicalTrials.gov: NCT04283461) and consists of a fragment of messenger RNA encapsulated in lipid nanoparticles, which once injected into the body, will express the viral antigen. Other approaches are in the pre-clinical phase and include subunitary vaccines based on recombinant proteins or viral vectors, attenuated or inactivated unit vaccines [127]. The SARS-CoV 2 vaccines must meet the minimum requirements to stimulate the specific immune response against the S protein, as well as the non-specific immune response (IFN synthesis). Adenoviruses (Adv) produce about 1/3 of all acute respiratory tract infections. The Adv5-vectored S vaccine induces anti-S and anti-N IgG synthesis and stimulates Th1 and Th2 lymphocytes in mice [132]. Anti-infectious protection of the digestive and respiratory epithelium is conferred by sIgA and TCD8 lymphocytes. The sIgA synthesis is relatively independent of the systemic immune response. Stimulation of TCD8 lymphocytes, essential for the effectiveness of anti-viral immunity, is dependent on DC’s ability to interact with Adv, making it available to the presentation of T lymphocytes.

If the Adv5-vectored S vaccine interacts with these components of the immune reactivity, hopes of limiting the SARS-CoV2 pandemic are high.

One of the biggest challenges with the SARS-CoV-2 vaccine is that the protective effect of the subunitary or attenuated vaccine will be temporary. SARS-CoV-2 undergoes mutations with a frequency almost as high as the influenza A virus, with an average of 1.60/10^3^ nucleotides/year. The accumulation of mutations in the SARS-CoV-2 genome over several seasons will probably generate a new antigenic variant, which will spread within the population lacking immune protection.

In any event, along with other preventive measures, a vaccine is the solution for which the greatest efforts are underway, given that this method of prophylaxis has proven effectiveness. These efforts will increasingly require the collaboration of members of the international scientific community and wider participation of the human community, for facing growing challenges.

## 10. Conclusions

Coronaviruses are large viruses that infect both animals and humans. Studies have shown that human transmission is possible both directly, through droplets, and indirectly by coming in contact with contaminated surfaces. These findings necessitate the implementation of additional safety measures, covering these possible alternative transmission routes. Regarding the IR in SARS-CoV-2 infection, in the early stages of infection, elimination of the SARS-CoV-2 from the respiratory tract is the result of the activation of a specific IR mediated by TCD4 and TCD8 lymphocytes. Subsequently, the severe clinical phase is characterized by a “cytokine storm”, in which neutrophils, eosinophils, macrophages, and CDs migrate into the infectious foci and release a cascade of pro-inflammatory cytokines. Since the onset of the pandemic, investigators have demonstrated that severe clinical forms of SARS-CoV-2 infection are associated with several risk factors such as old age, metabolic syndrome, diabetes, obesity, chronic inflammatory and autoimmune diseases, HT, smoking, etc. For confirming the SARS-CoV-2 diagnosis, the most commonly used method is the RT-PCR technique, owing to its high specificity. However, specialized techniques, trained staff, extended waiting periods, and other factors demonstrate the challenges associated with this technique. Rapid tests may be a more accessible alternative, but results obtained in several countries highlight the need to assess their accuracy. Both rapid and biochemical tests can be performed complementary with RT-PCR to obtain additional information regarding the patient’s immune status.

Efforts are currently being undertaken to develop efficient strategies for SARS-CoV-2, including the development of a vaccine. Currently, the most advanced vaccine under development is in Phase I clinical testing. Given the consequences of SARS-CoV-2 infection and the complexity of the host immune response, future studies are crucial to providing additional information regarding the onset of the IR and the relationship between SARS-CoV-2 and inflammation. Until the development of the vaccine, stopping inter-human transmission is very important but difficult to achieve, mainly due to the variable and long periods of time between the infective contact and symptoms’ occurrence, depending on the viral load, host immunity, age, and comorbidities.

## Figures and Tables

**Table 1:** Diagnostic tools for SARS-CoV 2 infection (adapted from Russo et al.).

Test	Specimen	Advantages	Limitations
Real time PCR	Nasopharyngeal and/or oropharyngeal swab, lower respiratory specimen	Currently, the gold standard High sensitivity and specificity	Requires special infrastructure and trained personnel Expensive Medium turnaround time (190 min) Incorrect sampling
RT-LAMP	Nasopharyngeal and/or oropharyngeal swab, lower respiratory specimen	Shorter turnaround time compared to RT-PCR (45-60 min) High sensitivity Can be used as substitute for RT-PCR when a reduced turnaround time is needed	Requires special infrastructure Incorrect sampling Expensive
Nucleoprotein antigen detection test	Nasopharyngeal and/or oropharyngeal swab, and/or lower respiratory specimen	Easier to use, suitable for labs which are less equipped	Low sensitivity Requires qualified personnel Incorrect sampling
ELISA	Serum, plasma, whole blood	Not very expensive Medium turnaround time	Requires special infrastructure and qualified personnel
Chemiluminescence immunoassay	Serum, plasma, whole blood	High sensitivity	Requires special infrastructure and qualified personnel
Rapid antibody (IgG and IgM) detection test	Fingerprick	Easy sampling Does not require special infrastructure Short turnaround time (max 30 min)	Low specificity and sensitivity More suitable for epidemiological screening rather than diagnosis per se

**Table 2:** Proposed drugs for the COVID-19 infection treatment

Drug	Mechanism of Action	Adverse Effects/Limitations	References
Chloroquine Hydroxychloroquine	Interferes with the terminal glycosylation of ACE2, and thus negatively influences the virus-receptor binding in SARS-CoV infection	Narrow therapeutic index Seizures Retinopathy Myopathy Bone marrow suppression	[99,100]
Remdesivir	An adenosine analog causing premature termination of the nascent viral RNA chains by incorporation into the viral genome	Kidney injury Elevated transaminases	[101]
Camostat/Nafamostat	TMPRSS2 inhibitors	Rash Diarrhea Nausea Hepatotoxicity	[102,103]
Imatinib	Abelson (Abl) kinase inhibitor, blocks the endocytic entry of other β-coronaviruses	GIT intolerance, Flu-like symptoms	[104]
Lopinavir & Ritonavir	Inhibits the activity of 3CLpro and is approved for the treatment of HIV/AIDS	GIT intolerance, vomiting, nausea Hepatotoxicity Pancreatitis	[105,106]
Arbidol	Inhibits virus entry/fusion of viral membranes with cellular membranes	Skin rash	[107]
Cyclosporin A	Approved immunosuppressant drug, interferes with protein interactions thereby affecting viral replication	nephrotoxicity, hypertension, increased blood urea nitrogen, increased serum creatinine,	[108]
Tocilizumab	Binds to both soluble and cell-associated IL-6R with high affinity. TCZ blocks IL-6 from initiating its pro-inflammatory downstream signaling, alleviating the host immune response	Hepatotoxicity Headache Hypertension Hematologic effects Increase in upper respiratory tract infections	[109]
Azithromycin	Inhibits protein synthesis in bacteria but harbors also anti-viral effects	QT interval prolongation	[110]
Favipiravir	Inhibits RNA-dependent RNA polymerase of RNA viruses which leads to chain termination	Diarrhea Neutropenia	[105]
Ribavirin	Nucleoside analog of guanosine inhibiting RNA polymerase and (chain terminator)	Hemolytic Anemia Teratogenic	[111]
Ivermectin	Anti-parasitic drug shown to inhibit replication of SARS-CoV-2 in vitro	Skin rash Muscle/joint pain	[112]
Immunoglobulin	Antibodies obtained from recovered patients	Headache Fever Malaise Thrombosis Renal impairment	[113,114]
Corticosteroids	Can reduce pathological damage caused by the infection harboring an anti -inflammatory role due to their various effects on various cytokines (1L-1, 1L-6, 1L-8, 1L-12, TNFα)	Long term use can cause; diabetes, hypertension, weight gain	[115]
Interferon	Used for boosting the immune system	Fever Chills Flu-like symptoms such as headache, fatigue, and weakness	[116]
CRISPR/Cas13d	Knockdown system used in cleaving the SARS-CoV-2 RNA genome; the Cas13d effector can be delivered via an adeno-associated virus (AAV) to the SARS-CoV-2 infected lung	Experimental, Expensive	[117]

**Table 3:** SARS-CoV-2 vaccine candidates currently in clinical trials.

Vaccine Name	Developer (Country)	Description	Clinical Trial Details
IMP (CoVac-1)	University Hospital Tuebingen (Germany)	Multipeptide cocktail; SARS-CoV-2 HLA-DR peptides, XS15 emulsified in Montanide ISA 51 VG	NCT04546841 Phase 1
TMV-083	Institut Pasteur (France)	Live-attenuated recombinant measles vaccine virus vector expressing a modified surface glycoprotein of SARS-CoV2	NCT04497298 Phase 1
EpiVacCorona	Federal Budgetary Research Institution State Research Center of Virology and Biotechnology “Vector” (Russia)	Chemically synthesized peptide antigens of SARS-CoV-2 proteins, conjugated to a carrier protein and adsorbed on an aluminum-containing adjuvant	NCT04527575 Phase 1
CoronaVac	Butantan Institute (Brazil) Sinovac Life Sciences Co., Ltd.	Adsorbed COVID-19 (inactivated) vaccine	NCT04456595 Phase 3
aAPC	Shenzhen Geno-Immune Medical Institute (China)	Coronavirus Artificial Antigen Presenting Cell Vaccine	NCT04299724 Phase 1
Gam-COVID-Vac	Gamaleya Research Institute of Epidemiology and Microbiology, Health Ministry of the Russian Federation (Russia)	adenoviral-based vaccine against SARS-CoV-2	NCT04436471
Inactivated SARS-CoV-2 vaccine (Vero cell)	China National Biotec Group Company Limited	Inactivated SARS-CoV-2 vaccine (Vero cell)	NCT04510207 Phase 3
Recombinant SARS-CoV-2 vaccine (Sf9 cell)	Jiangsu Province Centers for Disease Control and Prevention (China)	recombinant SARS-CoV-2 vaccine (Sf9 Cell)	NCT04530656 (Phase 1)
Lentiviral Minigene vaccine (LV-SMENP)	Shenzhen Geno-Immune Medical Institute	Lentiviral-SMENP-dendritic cell vaccine and antigen-specific CTLs	NCT04276896
UB-612	United Biomedical Inc., Asia	S1-RBD-protein-based vaccine	NCT04545749 Phase i
Covax-19™	GeneCure Biotechnologies	Therapeutic vaccine	NCT04428073
Recombinant Coronavirus-Like Particle Vaccine	Medicago	Recombinant Coronavirus-Like Particle	NCT04450004 Phase 1
mRNA-1273	ModernaTX, Inc	Lipid nanoparticle-incapsulated mRNA-based vaccine encoding the S protein of SARS-CoV2	NCT04283461 Phase 1
CTCOVID-19	CanSino Biologics Inc. (China)	Adenovirus Type 5 Vector	NCT04313127 Phase I
SCB-2019	Clover Biopharmaceuticals AUS Pty Ltd. (Australia)	Recombinant Trimeric S Protein Subunit Vaccine	NCT04405908 (Phase I)
BNT162b3	BioNTech RNA Pharmaceuticals GmbH (Germany)	Anti-viral RNA vaccine	NCT04537949 Phase 2
AZD1222	AstraZeneca	Non-replicating ChAdOx1 Vector vaccine	NCT04516746 Phase III
ChAdOx1	University of Oxford (UK)	chimpanzee adenovirus vaccine vector	NCT04324606
AG0302-COVID19	AnGes, Inc (Japan)	DNA vaccine	NCT04527081 Phase 2
CVnCoV	CureVac AG	mRNA vaccine	NCT04449276 Phase 1
Ad26.COV2.S	Janssen Vaccines & Prevention B.V.(Netherlands)	adenovirus serotype 26 (Ad26) vector-based vaccine	NCT04505722
AdimrSC-2f	Adimmune Corporation	the recombinant receptor-binding domain (RBD) of SARS-CoV-2 spike (S) protein amplified and purified using the baculovirus-insect cells expression system,	NCT04522089 Phase 1
GRAd-COV2	ReiThera Srl (Italy)	Encodes for SARS-COV-2 full length prefusion stabilized Spike protein gorilla-derived replication-defective adenoviral vector	NCT04528641 Phase 1
V-SARS	Immunitor LLC (Canada)	pill derived from heat-inactivated plasma from COVID-19 patient	NCT04380532 Phase 2
Spike nanoparticle with and without Matrix-M^TM^ adjuvant	Novavax (Australia)	Stable, pre-fusion spike nanoparticle with and without Matrix-M^TM^ adjuvant	NCT04368988 Phase 1 and 2
Inactivated SARS-CoV-2	Wuhan Institute of Biological Product (China)	Inactivated SARS-CoV-2	ChiCTR2000031809
PiCoVacc	Sinovac (China)	Inactivated SARS-CoV-2 (PiCoVacc) with an alum adjuvant	NCT04352608 Phase 2
Ad5-nCoV encoding full-length spike protein	CanSino Biologics (China)	Ad5-nCoV encoding full-length spike protein	NCT04341389 (Phase 2)
COVAC1	Imperial college London (UK)	mRNA SAM expressing spike protein in LNP	ISRCTN17072692 Phase 1
INO-4800	Inovio Pharmaceuticals (USA)	DNA expressing spike protein	NCT04336410 Phase 1
